# Aurin Tricarboxylic Acid Protects against Red Blood Cell Hemolysis in Patients with Paroxysmal Nocturnal Hemoglobinemia

**DOI:** 10.1371/journal.pone.0087316

**Published:** 2014-01-29

**Authors:** Moonhee Lee, Sujaatha Narayanan, Edith G. McGeer, Patrick L. McGeer

**Affiliations:** 1 Kinsmen Laboratory of Neurological Research, University of British Columbia, Vancouver, British Columbia, Canada; 2 Leukemia/BMT Program of BC, Division of Hematology, Vancouver General Hospital, BC Cancer Agency, Vancouver, British Columbia, Canada; Wake Forest Institute for Regenerative Medicine, United States of America

## Abstract

**Objectives:**

Paroxysmal nocturnal hemoglobinemia (PNH) is a rare but serious condition characterized by complement-mediated red blood cell (RBC) hemolysis and episodic thrombotic attack. It results from decay accelerating factor (CD55), and protectin (CD59), becoming attached to RBC and other cell surfaces. Absence of these protective proteins leaves such cells vulnerable to self attack at the C3 convertase and membrane attack complex (MAC) stages of complement activation. We have previously reported that aurin tricarboxylic acid (ATA) is an orally effective agent that selectively blocks complement activation at the C3 convertase stage as well as MAC formation at the C9 insertion stage.

**Design and Methods:**

We used a CH50 assay method and western blot analysis to investigate the vulnerability to complement attack of PNH RBCs compared with normal RBCs. Zymosan was used as the activator of normal serum and PNH serum. ATA was added to the sera to determine the concentration necessary to protect the RBCs from lysis by the zymosan-activated sera.

**Results:**

We found that erythrocytes from PNH patients on long term treatment with eculizumab were twice as vulnerable as normal erythrocytes to lysis induced by complement activated serum. Western blot data showed the presence of both C3 and C5 convertases on the PNH patient erythrocyte membranes. These data indicate persistent vulnerability of PNH erythrocytes to complement attack due to deficiencies in CD55 and CD59. ATA, when added to serum in vitro, protected PNH erythrocytes from complement attack, restoring their resistance to that of normal erythrocytes.

**Conclusions:**

We conclude that ATA, by protecting PNH erythrocytes from their decay accelerating factor (CD55) and protectin (CD59) deficiencies, may be an effective oral treatment in this disorder.

## Introduction

Paroxysmal nocturnal hemoglobinemia (PNH) is an episodic disorder involving complement-mediated hemolytic anemia, with an accompanying risk of thrombosis [1], [2]. PNH is a rare disease that was first recognized in the second half of the nineteenth century. However it was not properly understood until investigators discovered that PNH patients develop stem cell clones in their marrow that have a deletion of glycosyl phosphoinositol (GPI)-anchored proteins (GPI-APs) [3]. Genetic studies have identified the cause to be somatic mutations in the gene phosphatidylinositol glycan class A (PIG-A) [4], [5]. The gene encodes enzymes catalysing the first step of GPI-anchor-biosynthesis, in which there is a transfer of N-acetylglucosamine to phosphatidylinositol in hematopoietic stem cells [4], [5]. The proteins which fail to become anchored, and are therefore non-functional, include decay-accelerating factor (DAF, CD55), an inhibitor of alternative pathway C3 convertase, and protectin (CD59), an inhibitor of membrane attack complex (MAC) formation [6]–[8].

Treatment of PNH has been considerably advanced by the introduction of eculizumab. It is a humanized monoclonal antibody derived from a murine anti C5 antibody, which binds to C5 and prevents C5 cleavage by C5 convertase. It inhibits red blood cell (RBC) lysis by limiting the amount of C5 available for MAC synthesis [9]. Long term treatment of PNH cases with biweekly intravenous infusions of eculizumab has been reported to restore normal life expectancy, and, in two thirds of patients, to eliminate the need for transfusions [10], [11]. It is not totally effective since it does not compensate for the lack of CD55 on erythrocytes [12]. Treatment with eculizumab enhances survival of CD55 deficient erythrocytes, rendering them sensitive to subsequent hemolysis. This helps to explain the continuing vulnerability of some PNH patients to hemolytic attack, the need for transfusions, and a continuing risk of thrombosis [12].

Previously we reported that aurin tricarboxylic acid (ATA) inhibits both the classical and alternative complement pathways by blocking C9 addition to C5b-8, thus inhibiting MAC formation [13]. We have further reported that ATA inhibits the C3 convertase step in the alternative pathway by blocking factor D cleavage of membrane bound factor B in the complex properdin-C3b-factor B (PC3bB) [14]. Thus it inhibits both C3 convertase as well as MAC formation.

In the present investigation, we evaluated the potential effectiveness of ATA as a treatment for PNH by studying the red blood cells (RBCs) and serum from 5 PNH patients on eculizumab therapy. Samples were taken just prior to their biweekly infusion. We found that the RBCs from PNH patients, at the time of infusion, were not completely protected by eculizumab from complement attack. Modest levels of ATA added to PNH serum, which had been supplemented with C5 to compensate for eculizumab, fully restored the PNH RBC protection. This suggests that ATA may be an effective treatment for PNH.

## Methods

### Patient Selection

Five patients being treated with eculizumab for PNH at the Vancouver General Hospital were selected for this study. All were on a maintenance dose of 900 mg intravenously every two weeks. The blood samples and related clinical data used in this project were provided by the Hematology Cell Bank of British Columbia with Research Ethics Board Approval (No: H04-61292). Written consent was obtained from each patient. The consent procedure was approved by the University of British Columbia Clinical Research Ethics Board. Two blood tubes were taken just prior to analysis. One contained no additive so the blood could be allowed to clot and the serum harvested. The other contained the standard hospital anticoagulant EDTA and was used to harvest RBCs. All the patients had received vaccination against *Neisseria meningitides* around the time of commencement of eculuzimab. Four of the five patients are also currently on Penicillin V prophylaxis as the commercially available vaccine does not provide protection against Group B meningococcus.

Patient 1 is a 39 year old man diagnosed with PNH. The patient suffered from intermittent episodes of hemolysis associated with abdominal pain on a background of chronic hemolysis. The PNH clone size was quantitated at 90% (type III) measured in 2003 by flow cytometry in the neutrophil and monocyte series. He commenced treatment with eculizumab 3 years ago and since then has been relatively stable, with significant reductions in symptoms and acute hemolytic episodes. Two blood samples were obtained from this patient approximately one year apart (1–1 and 1–2 in tables).

Patient 2 is a 24 year old man who was diagnosed with PNH as an 11 year old following investigation for a mild pancytopenia. His PNH type III clone was noted to be 60% by flow cytometry. He remained reasonably stable until July 2010 when he presented with a protracted episode of intravascular hemolysis following a urinary tract infection with *Streptococcus viridans*. This was followed by recurrent episodes of acute hemolysis associated with abdominal pain without significant precipitating factors warranting blood transfusions every 4–6 weeks. His PNH clone rose to 90% and he developed a calf vein deep vein thrombosis. Two years ago he commenced eculizumab with no further episode of acute hemolysis or thrombosis. He is transfusion independent.

Patient number 3 is a 43 year old lady who presented 9 years ago with dysfunctional uterine bleeding and pancytopenia. She was diagnosed with severe aplastic anemia (SAA) and a PNH clone of 10% was noted. Her SAA was treated with horse anti-thymoglobulin (ATG) and cyclosporine (CSA). Four years ago, when her PNH clone increased to 60% (Type III), she developed pancytopenia. A bone marrow biopsy confirmed moderate erythroid dysplasia but normal megakaryocytic and granulocytic lineage with a normal karyotype. She developed repeated episodes of intravascular hemolysis three years ago and was started on eculizumab. This significantly improved hemolysis but she has remained pancytopenic requiring intermittent red cell transfusions.

Patient 4 is a 27 year old man who presented three years ago with hemoglobinuria associated with gastroenteritis. Investigation revealed a pancytopenia with a PNH type III clone of 93%. He required blood transfusions on an ongoing basis. Eight months ago he commenced eculizumab which improved the hemolysis. However he developed a progressive pancytopenia. He was diagnosed with a myelodysplastic/myeloproliferative neoplasm and has undergone a sibling myeloablative stem cell transplant.

Patient 5 is a 30 year old lady who presented 12 years ago with a pancytopenia. She was diagnosed with SAA which was treated with ATG/CSA. Over subsequent years she was troubled by recurrent episodes of intravascular hemolysis requiring hospital visits. Her PNH clone (type III) has been quantitated to be 76–86% by flow cytometry. She was commenced on eculizumab two and a half years ago and has since then been stable with no further hemolytic episodes. Two blood samples were obtained from this patient approximately one year apart (5-1 and 5-2 in tables).

Our small patient series demonstrates the high variablility of abnormal clones that may be encountered both within and between PNH cases. They ranged from 10% to 93%. Such variability has previously been noted [15].

### Blood Treatment and CH50 Type Assays

Blood samples in the normal tubes were treated at 37°C for 3 h to promote clotting. Samples were then centrifuged at 3,000 g for 10 min and the clear serum harvested. Blood samples in the EDTA tubes were directly centrifuged at 3,000 g for 10 min. An equal volume of Hank’s balanced salt solution (HBSS) was added to the pellet and the red blood cells (RBCs) re-suspended. The mixture was centrifuged as before and the washed RBCs harvested.

For hemolysis assays, a CH50 type system was employed. Zymosan (Sigma, St. Louis, MO) was utilized as the serum complement activator after initial purification. It was first dissolved in Hank’s balanced salt solution (HBSS) at a concentration of 1 µg/ml, vigorously shaken at 37°C for 1 h, and then centrifuged at 7,400 g for 5 min. The purpose was to remove the large particles so that satisfactory western blot results could be obtained. The small particles remaining in the supernatants were fully capable of activating serum complement, and were used for all experiments. The standard reaction mixture contained 50 µl of RBCs (5×10^6^ cells) in HBSS, 50 µl of zymosan (1 µg/ml), 25 µl of serum, and a further 25 µl of HBSS for a final volume of 150 µl. The mixtures were incubated at 37°C for 1 h and were then centrifuged at 3,000 g for 10 min. The supernatants (100 µl) were transferred to 96 well ELISA plates and the optical density (OD) at 405 nm read with an ELISA reader (Bio-Rad, CA). The RBC pellets were utilized for further experiments.

### Western Blot Analysis

Western blot analysis was performed as described by Lee et al. [14]. Briefly, the RBCs from PNH patients (5×10^6^ cells) were directly treated with sample loading buffer. The buffer consisted of 50 mM Tris (pH 6.8), 0.1% SDS, 0.1% bromophenol blue and 10% glycerol. For positive controls, RBCs from normal controls were treated with normal human serum plus zymosan and incubated at 37°C for 1 h. The reaction mixtures were centrifuged at 30,000 g for 3 h and the supernatants discarded. The precipitates were treated with sample loading buffer. For negative controls, normal human serum and RBCs were directly treated with sample loading buffer. An untreated volume of 25 µl from each sample was directly loaded onto 12% polyacrylamide gels and separated under non-reducing conditions (70 V, 3–6 h). Following SDS-PAGE, proteins were transferred to a PVDF membrane (Bio-Rad, CA) at 30 mA for 6 h. The membranes were blocked with 5% milk in PBS-T (80 mM Na_2_HPO_4_, 20 mM NaH_2_PO_4_, 100 mM NaCl, 0.1% Tween 20, pH 7.4) for 1 h, and incubated overnight at 4°C with a primary antibody to C3/C3b (1/2,000, Quidel, San Diego, CA), C4/C4b (1/2,000, Abcam, Cambridge, MA) and properdin (1/2,000, Quidel, San Diego, CA). The membranes were then treated with horseradish peroxidase (HRP)-conjugated anti mouse IgG antibody (A3682, Sigma, 1/3,000) for 3 h at room temperature. The bands were visualized with an enhanced chemiluminescence system and exposure to photographic film (Hyperfilm ECL™, Amersham Pharmacia Biotech, Little Chalfont, UK). For reprobing of membranes with different antibodies, the membranes were treated with 10 ml of stripping buffer (3 ml of 1 M Tris-HCl pH 6.7, 10 ml of 10% SDS, 347 µl of 14 M β-mercaptoethanol, and 36.5 ml deionized water) at 60°C for 1 h. The membrane to be reprobed was then placed on a shaking incubator for 30 min to clear it of all the bound antibodies. The stripped membrane was then treated with a different primary antibody.

To investigate directly whether ATA inhibits formation of C3 convertase and the MAC on PNH RBCs exposed to zymogen activated PNH serum, additional CH50 assays were carried out on each PNH patient with and without the addition of 1.67 µM ATA. C1 inhibitor (1.8 µg/ml) was added to block classical pathway activation. Cells were separated and western blotting carried out on the RBC membranes as described previously. Primary antibodies for immunoblotting were polyclonal goat anti C6 (1/2,000, Quidel) to detect the MAC or monoclonal mouse anti C3/C3b (1/2,000, Quidel) to detect C3 convertase. Secondary antibodies were HRP-conjugated anti goat IgG (1/3,000, Millipore, Billerica, MA) and HRP-conjugated anti mouse IgG (1/3,000, Sigma). For control purposes, comparative western blots were carried out on 5 cases using normal RBCs exposed to zymogen activated normal serum.

### Data Analysis

Data were analyzed using microcal origin version 6.0 (Origin Lab Corporation, Northampton, MA) and are expressed as mean±SEM. The dose-response curves were fitted as first order exponential decay. The significance of differences was analyzed by one-way and two-way ANOVA tests. Multiple group comparisons were followed by a *post-hoc* Bonferroni test.

## Results

Firstly, we used our standard CH50 assay to compare the vulnerability of RBCs from PNH patients with RBCs from normals. For this purpose, sera from normals and sera from PNH patients were exposed to zymosan-activated sera. PNH sera were tested with and without C5 added. ATA was added in some experiments to evaluate its effects in blocking complement activation. For comparative purposes, a standard Ham assay was also run on all patients’ RBCs. Almost identical results were obtained to the CH50 assays (data not shown).

**Figure 1:**
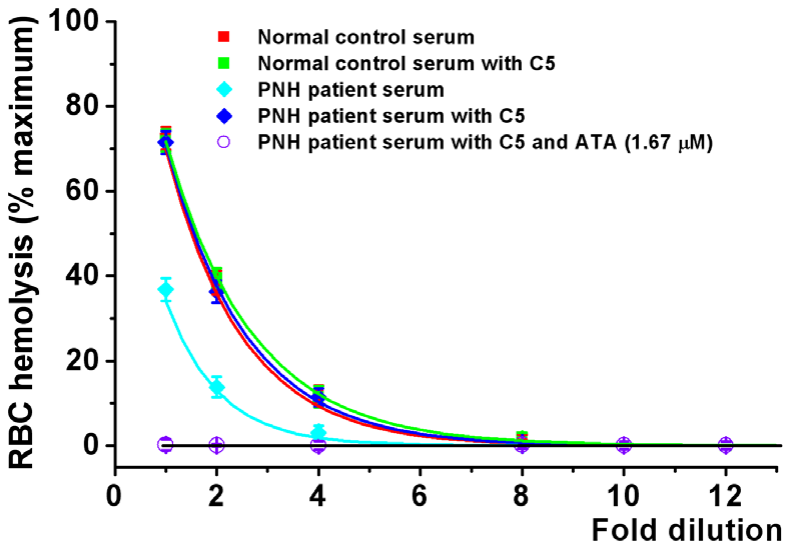
Normal and PNH red blood cell (RBC) lysis by 1–12 fold dilutions of normal and PNH serum. Serum complement was activated by zymosan. Note that PNH serum, which contained eculizumab, was less active than normal serum. However addition of C5 protein at a final concentration of 100 µg/ml increased PNH RBC lysis to the same levels as control RBCs. Most importantly, ATA at a concentration of 1.67 µM completely blocked PNH RBC lysis. Values are mean±SEM, n = 4. Significance of differences was tested by two-way ANOVA. Multiple comparisons were followed with *post-hoc* Bonferroni tests where appropriate. The significance was p<0.01 for PNH patient serum with C5 added compared with PNH patient serum without C5 added, and p<0.01 for PNH patient serum with and without C5 compared with PNH patient serum plus ATA.

Figure 1 shows typical results of the amount of hemoglobin released from lysed RBCs as a function of complement activated serum dilution. Figure 1 shows that, under the conditions of the experiment, 70% of normal RBCs were lysed to release their hemoglobin when they were exposed to undiluted normal serum that had been activated by zymosan. The total hemoglobin available was determined by lysing 100% of the RBCs with lysis buffer. No lysis was observed when ATA was added to the serum at a final concentration of 1.67 µM. This establishes that ATA gave complete protection to normal RBCs at this concentration. Without ATA, the lysis decreased in a dilution-dependent manner. Analysis of the 7 normal samples showed the average RBC lysis with undiluted serum was 71.86%, with a serum dilution to eliminate RBC lysis of 10.17 fold (Table 1).

**Table 1:** Comparison of RBC lysis (% of maximum available) in undiluted and diluted serum.

	Normal control		PNH patients	
Patient identification	RBC lysis (%) no dilution	Dilution fold to eliminate RBC	RBC lysis (%) no dilution	Dilution fold to eliminate RBC lysis
1-1	72	10.5	35	5.2
2	75	10.2	53	7.3
3	70	9.4	30	4.1
4	72	11.1	37	3.9
5-1	71	10.2	38	4.8
1-2	70	10.1	35	5.2
5-2	73	9.7	38	4.6
Mean	71.86	10.17	38	5.01

The dilution fold of serum is that which completely eliminated RBC lysis. Repeat samples of patients 1 and 5 are designated as 1-2 and 5-2.

Differing results were obtained from the PNH patients. They were all on chronic eculizumab treatment. Their sera partially inhibited zymosan activation by blockade of endogenous C5. Instead of 70% hemolysis, zymosan activated sera from the PNH patient caused lysis of only about 35% of the patient’s RBCs (Table 1). This was also reduced in a concentration-dependent manner so that there was no RBC lysis with a 5.2-fold serum dilution. Analysis of the 7 PNH samples showed the average RBC lysis with undiluted serum was 38%, with a serum dilution to eliminate RBC lysis of 5.01 fold (Table 1).

When C5 was added to the patient’s serum, it regained a full capacity to lyse the patient’s RBCs. To be certain that sufficient C5 was available, we added it at a concentration of 100 µg/ml, since that exceeds the normal C5 concentration of about 75 µg/ml. It was found that adding C5 protein at this concentration increased RBC lysis by PNH serum to equivalent levels of normal serum. However, as expected, adding C5 protein to normal serum had no effect on RBC lysis (Figure 1).

**Figure 2:**
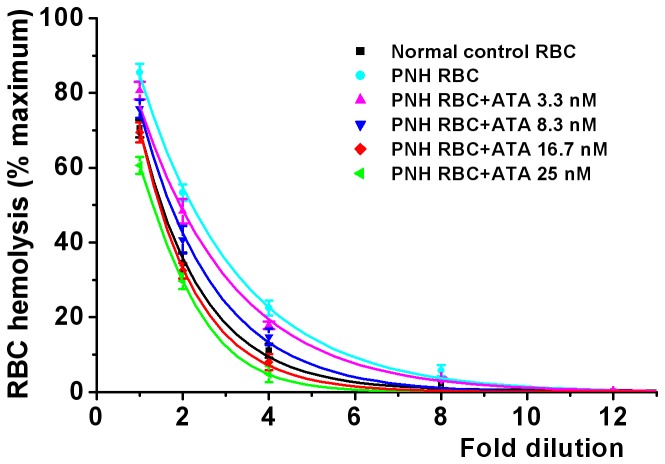
Effect of various concentrations of ATA in providing protection of patient 1 PNH RBCs to lysis by zymosan activated serum. ATA-treated PNH RBCs (3.3 nM, 8.3 nM and 25 nM) were compared with normal RBCs at 1 to 8 fold serum dilutions. ATA provided protection in a concentration dependent manner, achieving equality with normal RBCs at a concentration of 15.1 nM. Values are mean±SEM n = 4, p<0.01 Significance of differences was tested by two-way ANOVA. Multiple comparisons were followed with *post-hoc* Bonferroni tests where appropriate.

The next set of experiments was designed to determine the minimum concentration of ATA required to provide complete protection of PNH RBCs. In these experiments, ATA at final concentrations of 3.3, 8.3, 16.7 and 25 nM was added to zymosan-activated PNH serum that had been supplemented with C5 at a concentration of 100 µg/ml. Comparisons were made with normal RBCs. A typical result is shown in Figure 2. It was found that zymosan-activated normal serum hemolyzed 70% of healthy RBCs (black lines in Figure 2) and 86% of PNH RBCs (light blue lines). There was no hemolysis at an 8 fold serum dilution of normal RBCs and at a 12 fold dilution of PNH RBCs. ATA supplementation reduced PNH RBC lysis in a concentration-dependent manner. The sensitivity of PNH RBC lysis was neutralized to that of normal RBCs at an ATA concentration of 16.7 nM (red lines). Analysis of the results from the 7 serum samples is shown in Table 2. The mean ATA concentration to achieve complete protection was 10.59 nM.

**Table 2:** ATA concentrations required to provide protection of PNH RBCs to normal RBC levels from zymosan activated serum.

Patient Identification	ATA concentration for neutralization (nM)
1-1	15.1
2	8.3
3	8.3
4	5
5-1	9.93
1-2	16.7
5-2	10.83
Mean	10.59

**Figure 3:**
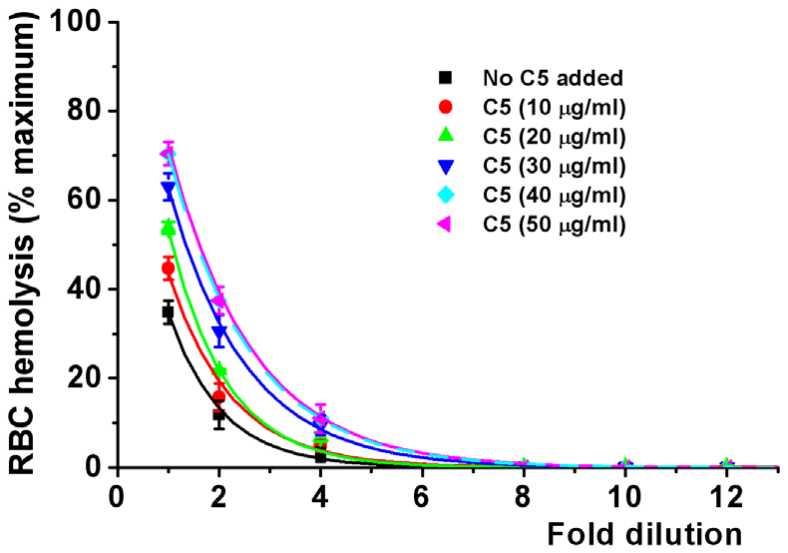
Effect of various concentrations of C5 protein added to PNH serum to neutralize the eculizumab blockade. PNH and normal RBCs were tested against zymogen activated serum. C5 protein addition increased the sensitivity of PNH RBCs to lysis in a concentration-dependent manner. The level needed to overcome the block and reach normal RBC sensitivity was 40 µg/ml. Values are mean±SEM, n = 4, Significance of differences was tested by two-way ANOVA. Multiple comparisons were followed with *post-hoc* Bonferroni tests where appropriate. p<0.01 for C5-treated PNH serum compared with untreated serum.

A further set of experiments was carried out to determine how much C5 was needed to restore the PNH patients’ sera to full complement capacity. The purpose was to determine how much residual C5 activity was available for complement attack at the time of eculizumab reinfusion. A typical result is shown in Figure 3. The PNH serum hemolyzed 34% of PNH RBCs (black lines). Addition of C5 proteins increased RBC hemolysis, which was concentration-dependent. Maximal hemolysis was shown with addition of 40 µg of C5 protein (light blue lines). The full results are shown in Table 3. Analysis of the 7 samples showed that the mean C5 protein required to restore the full complement activation capacity was only 37.57 µg/ml. Of course this amount would be larger immediately following an eculizumab infusion.

**Table 3:** Amount of C5 protein needed to overcome eculizumab block of PNH RBC lysis.

Patient Identification	C5 protein added to overcome eculizumab block (µg)
1-1	40
2	36
3	40
4	42
5-1	29
1-2	45
5-2	31
Mean	37.57

The next set of experiments investigated whether PNH patients have C3b and C5b fragments on their RBC membranes, which would be indicative of persistant opsonization. For these experiments, RBC membranes from PNH patients were directly treated with sample loading buffer and western blot analysis of the dissolved samples performed as described under methods [15]. Blots were treated with monoclonal antibodies to C3/C3b, C4/C4b and properdin. Normal serum and normal RBCs were used as controls.

**Figure 4:**
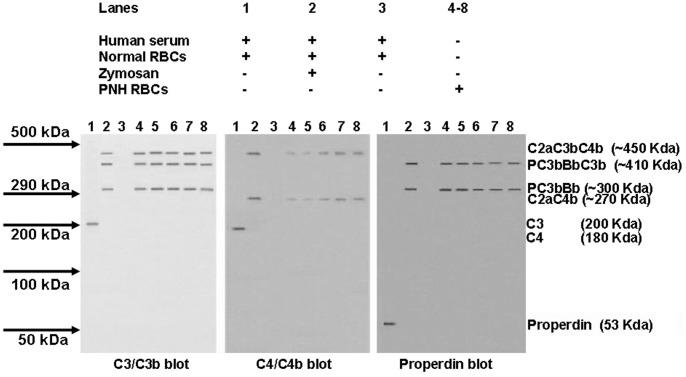
Western blot analyses of C3b, C4b and properdin complexes of RBCs from five PNH patients (lanes 4–8) compared with normal RBCs (lanes 1–3). Normal human serum was utilized in lanes 1–3, with zymosan activated serum being added in lane 2. See methods for details. Three independent experiments were performed and these are representative blots. Normal serum (lane 1) in the presence of normal RBCs contained detectable levels of full length C3, C4, and properdin. When serum was activated with zymogen (lane 2) normal and PNH RBCs showed bands corresponding to C2aC3bC4b and PC3Bb when developed with a C3 antibody, C2aC3bC4b and C2a4b when developed with a C4 antibody, and PC3Bb when developed with a properdin antibody. These data show that both the classical and alternative pathways have been activated on the surface of RBCs by zymogen treatment. Note that there were C3 convertases (C2aC4b and PC3bBb) and C5 convertases (C2aC3bC4b and PC3bBbC3b) on the surface of PNH RBCs despite eculizumab treatment. Normal RBCs were first solubilized with sample loading buffer. PNH RBCs were directly treated with sample loading buffer.

Results are shown in Figure 4. Untreated serum (lane 1) showed single bands corresponding to C3 and C4 and properdin. Lane 2, in which zymosan treated serum was added to normal RBCs, showed bands corresponding roughly in MW to C2aC3bC4b (450 kDa), PC3bBbC3b (410 kDa), PC3bBb (300 kDa) and C2aC4b (270 kDa). It must be noted that C3b and C4b proteins bind covalently to entities on human RBC membranes so the bands probably include unidentified entities such as glycophorins derived from the RBC membranes themselves. These bands indicate that C3 had been cleaved and attached to RBC membranes by both classical and alternative pathway activation. Lane 3 shows that no bands were visible on normal RBC membranes that had not been exposed to complement activation by zymosan.

Lanes 4–8 show the RBC results from five PNH patients. Each sample showed strong bands corresponding to C2aC3bC4b, PC3bBbC3b, PC3bBb and C2aC4b. These results demonstrate that in all five cases, the RBCs had been exposed to complement activation by both the classical and alternative pathways. The results show that C3 and C5 convertases were present on their RBCs in vivo. This result would be expected on the basis of a deficiency in CD55.

**Figure 5:**
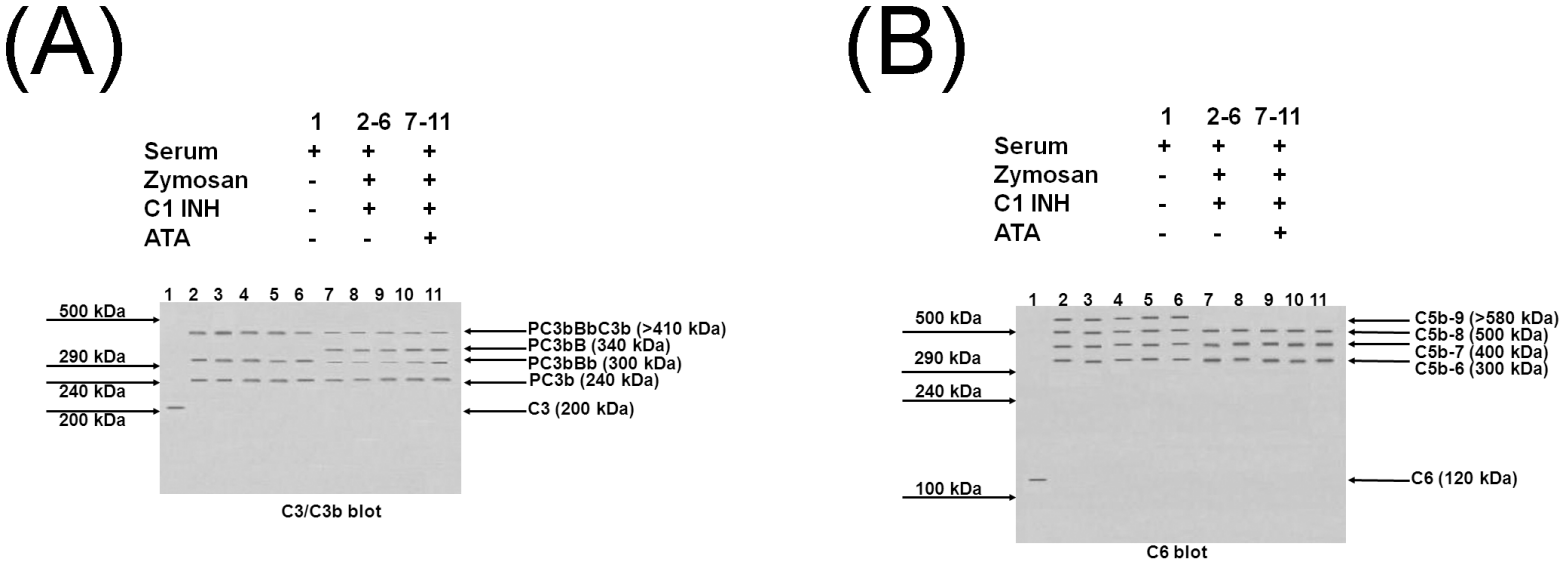
Western blot analyses of PNH patient RBCs exposed to their corresponding serum. Serum was activated by zymogen in the presence of C1 inhibitor. Figures 5A shows the results when the membrane was developed with the C3/C3b antibody and Figure 5B when it was developed with the C6 antibody. See methods for details. Lane 1 PNH serum; lanes 2–6 patient RBCs exposed to complement activated serum; lanes 7–1l corresponding lanes to 2–6 plus addition of ATA. Notice in lanes 2–6 of 5A the presence of bands corresponding in MW to PC3b, PC3bBb, and PC3bBbC3b indicating that C3 convertase had been activated, and in lanes 7–11, a new band for PC3bB with attenuation of the bands for PC3bBb and PC3bBbC3b indicating a strong reduction in C3 convertase on the RBC membranes. Figure 5B shows bands in lanes 2–6 corresponding in MW to C5b6, C5b67, C5b678, and C5b6789, the latter corresponding to the MAC. These data indicate that, despite the presence of eculizumab in the serum, there was sufficient unbound C5 to permit the MAC to form. Lanes 7–11 show that in the presence of ATA, assembly was halted at the stage of C5b678. MAC formation was completely blocked indicating that ATA had bound strongly to C9 in the serum.

The final set of experiments was to determine directly whether ATA inhibits formation of C3 convertase and the MAC on PNH RBCs exposed to zymogen activated PNH serum. CH50 assays were carried out on each PNH patient with and without the addition of 1.67 µM ATA. C1 inhibitor (1.8 µg/ml) was added to block classical pathway activation. Figure 5A shows the western blotting results when the membrane was developed with the C3/C3b antibody and Figure 5B when it was developed with the C6 antibody. Lane 1 is PNH serum. Lanes 2–6 are patient RBCs plus their corresponding serum with zymosan added to activate complement and C1 inhibitor added to block the classical pathway. Lanes 7–11 correspond with lanes 2–6 except that ATA has been added as a protective agent. Lanes 2–6 of Figure 5a show that each patient RBC demonstrated bands corresponding in MW to PC3b, PC3bBb, and PC3bBbC3b indicating that C3 convertase had been activated on the RBC surfaces. Lanes 7–11, where ATA had been added, show strong bands for PC3bB appearing with attenuation of the bands for PC3bBb and PC3bBbC3b. These results indicate that factor D, which cleaves membrane bound PC3bB, has been severely inhibited by ATA, indicating a strong reduction in C3 convertase. Figure 5B, developed by C6, shows in lanes 2–6 bands corresponding in MW to C5b6, C5b67, C5b678, and C5b6789, the MAC. These data indicate that, despite the presence of eculizumab in the serum, there was sufficient unbound C5 to permit the MAC to form. Lanes 7–11 show that in the presence of ATA, assembly was halted at the stage of C5b678. MAC formation was completely blocked indicating that ATA had bound strongly to C9 in the serum.

## Discussion

The data of this investigation showed that PNH RBCs were twice as vulnerable as normal RBCs to complement attack (Figure 1 and Table 1). A significant degree of protection was given to the PNH patients by eculizumab. At the time of eculizumab readministration, that protection was not total since hemolysis of 38.6% of PNH RBCs could still be induced by activating their serum with zymosan. However ATA, at a concentration of 1.67 µM, gave complete protection even when the PNH serum had been fortified with excess C5 (Figure 1). Titration of the amounts of ATA which were actually required to restore full protection averaged only 10.59 nM (Figure 2 and Table 2).

The amounts of C5 required to restore PNH serum to its full capacity to activate complement were also considerably less than the 100 µg/ml. As illustrated in Figure 3 and Table 3 the actual amounts required to restore full activity averaged only 37.57 µg/ml. The values reflect the state of affairs just before eculizumab infusion and would be expected to rise after the infusion.

As for the patient RBCs, our western blot data show that C3 and C5 convertases were present on the membranes of all five PNH cases, and that they had been exposed to complement activation of both the classical and alternative pathways (Figure 4) despite eculizumab treatment. It has previously been suggested that there is an actual increase in their RBC sensitivity due to such treatment because of a prolongation of abnormal RBC survival [12], [13]. Our result is consistent with a previous report that all patients on eculizumab had C3 bound on their RBCs surface [12]. Eculizumab can compensate for the C5 convertase activation, but not for the C3 convertase activation. Some PNH patients are known to suffer from persistant anemia. This is consistent with reports that clones of PNH RBCs lacking the protection of CD55 can dominate the total population of RBCs in peripheral blood [12]. ATA, by blocking both C3 convertase and MAC formation, can compensate for PNH deficiencies in both CD55 and CD59.

Figure 5 shows that, despite eculizumab treatment, zymogen activation of complement in the serum of PNH patients will still result in some activation of C3 convertase on their RBC membranes, as well as deposition of the MAC. This potential for in vivo damage can be prevented by ATA.

We have previously shown in the B6SJL-Tg mouse model of Alzheimer disease, that ATA is a non-toxic orally effective agent [13]. When provided chronically as a food supplement to these mice, it was without apparent toxicity. The mouse serum had a 3.59 fold protection against complement mediated RBC hemolysis compared with serum from mice fed non-supplemented chow, demonstrating serum protection following oral administration.

Our study shows that ATA can provide protection in the nanomolar concentration range (Figure 2 and Table 2). We note here that all 5 PNH patients were being treated with infusions of eculizumab every two weeks. ATA could be an alternative treatment where pills, capable of maintaining a steady state of MAC inhibition, could be taken every day.

A concern with ATA treatment, as well as with eculizumab administration, is the possibility of neisseria infection. It has been reported that 50–60% of individuals with a deficiency in the late-complement components will experience at least one episode of meningococcal disease and that they are 7,000–10,000 times more vulnerable to such an infection than the normal population [16]. It is difficult to reconcile such a report when it is known that 0.12% of Japanese [17] and 0.049% of Koreans [18] have a complete deficiency in C9. While there have been rare reports of meningococcal infections in C9 deficient Japanese, it is noted that the vast majority are usually healthy [19]. With respect to eculizumab treatment, precautionary action was taken by immunizing all patients. The same could be done with ATA administration.
